# Model of metabolism and gene expression predicts proteome allocation in *Pseudomonas putida*

**DOI:** 10.1038/s41540-025-00521-1

**Published:** 2025-05-24

**Authors:** Juan D. Tibocha-Bonilla, Vishant Gandhi, Chloe Lieng, Oriane Moyne, Rodrigo Santibáñez-Palominos, Karsten Zengler

**Affiliations:** 1 Bioinformatics and Systems Biology Graduate Program, University of California, San Diego, 9500 Gilman Drive, La Jolla, CA 92093-0760 USA; 2 Department of Bioengineering, University of California, San Diego, La Jolla, CA 92093-0412 USA; 3 Department of Pediatrics, University of California, San Diego, 9500 Gilman Drive, La Jolla, CA 92093-0760 USA; 4 Center for Microbiome Innovation, University of California, San Diego, 9500 Gilman Drive, La Jolla, CA 92093-0403 USA; 5 Program in Materials Science and Engineering, University of California, San Diego, 9500 Gilman Drive, La Jolla, CA 92093-0418 USA

**Keywords:** Biochemical networks, Biotechnology, Systems biology

## Abstract

The genome-scale model of metabolism and gene expression (ME-model) for *Pseudomonas putida* KT2440, *i*Ppu1676-ME, provides a comprehensive representation of biosynthetic costs and proteome allocation. Compared to a metabolic-only model, *i*Ppu1676-ME significantly expands on gene expression, macromolecular assembly, and cofactor utilization, enabling accurate growth predictions without additional constraints. Multi-omics analysis using RNA sequencing and ribosomal profiling data revealed translational prioritization in *P. putida*, with core pathways, such as nicotinamide biosynthesis and queuosine metabolism, exhibiting higher translational efficiency, while secondary pathways displayed lower priority. Notably, the ME-model significantly outperformed the M-model in alignment with multi-omics data, thereby validating its predictive capacity. Thus, *i*Ppu1676-ME offers valuable insights into *P. putida*’s proteome allocation and presents a powerful tool for understanding resource allocation in this industrially relevant microorganism.

## Introduction

*Pseudomonas putida* KT2440 is a versatile and metabolically robust biotechnological workhorse^1,2^. It thrives in diverse environments^3,4^, degrades a wide range of organic compounds^4,5^, and as a result, is employed to produce a variety of bulk and fine chemicals^6^. Recent studies have focused on harnessing the potential of *P. putida* KT2440 and on understanding and manipulating its metabolic network^7^. Genome-scale metabolic models (M-models) have long been used in metabolic engineering to identify metabolic bottlenecks and potential improvements in metabolic pathways for bioproduction^8–10^. While M-models provide valuable insights into metabolic capabilities, they do not account for macromolecular expression and the biosynthetic cost of enzymes and, as a consequence, require extensive constraining^11–13^. Thus, predictions using M-models can lack robustness^11^, making it challenging to predict engineering strategies to improve performance^14^.

Models of metabolism and gene expression (ME-models) mechanistically describe gene expression pathways and their intertwined role with metabolic pathways to achieve optimal resource allocation for growth^12^. As a result, ME-models make predictions beyond the scope of traditional M-models, including unconstrained by-product secretion^11^, overflow metabolism^15^, cofactor usage^15^, protein overproduction^14^, and proteomic responses to stress conditions^16^. However, the reconstruction of ME-models is time-intensive and requires extensive manual curation, which has led to a reduced number of reconstructed ME-models, only available for *Bacillus subtilis*^14^, *Clostridium ljungdahlii*^15^, *Escherichia coli*^12,17^, and *Thermotoga maritima*^11^.

Here, we reconstructed an ME-model for *P. putida* KT2440, *i*Ppu1676-ME, offering an unprecedented level of detail in the cellular function and proteome allocation of this bacterium. We show the improved predictive capabilities of proteome limitation in *P. putida* KT2440. Furthermore, we interrogated the gene expression of *P. putida* KT2440 using transcriptomics (RNA-Seq) and translatomics (Ribo-Seq) data. We analyzed the translational prioritization of pathways, as well as pathways that were significantly less prioritized for translation. When contrasting the model predictions against these sequencing datasets, we found stronger agreement with the ME-model compared to the M-model. Thus *i*Ppu1676-ME represents a valuable asset for accurate predictive modeling as a tool for bioprocess design and optimization and metabolic engineering^8–10^ in this industrially important strain.

## Results

### *i*Ppu1676-ME predicts proteome limitation and overflow metabolism in *P. putida* KT2440

**Fig. 1:**
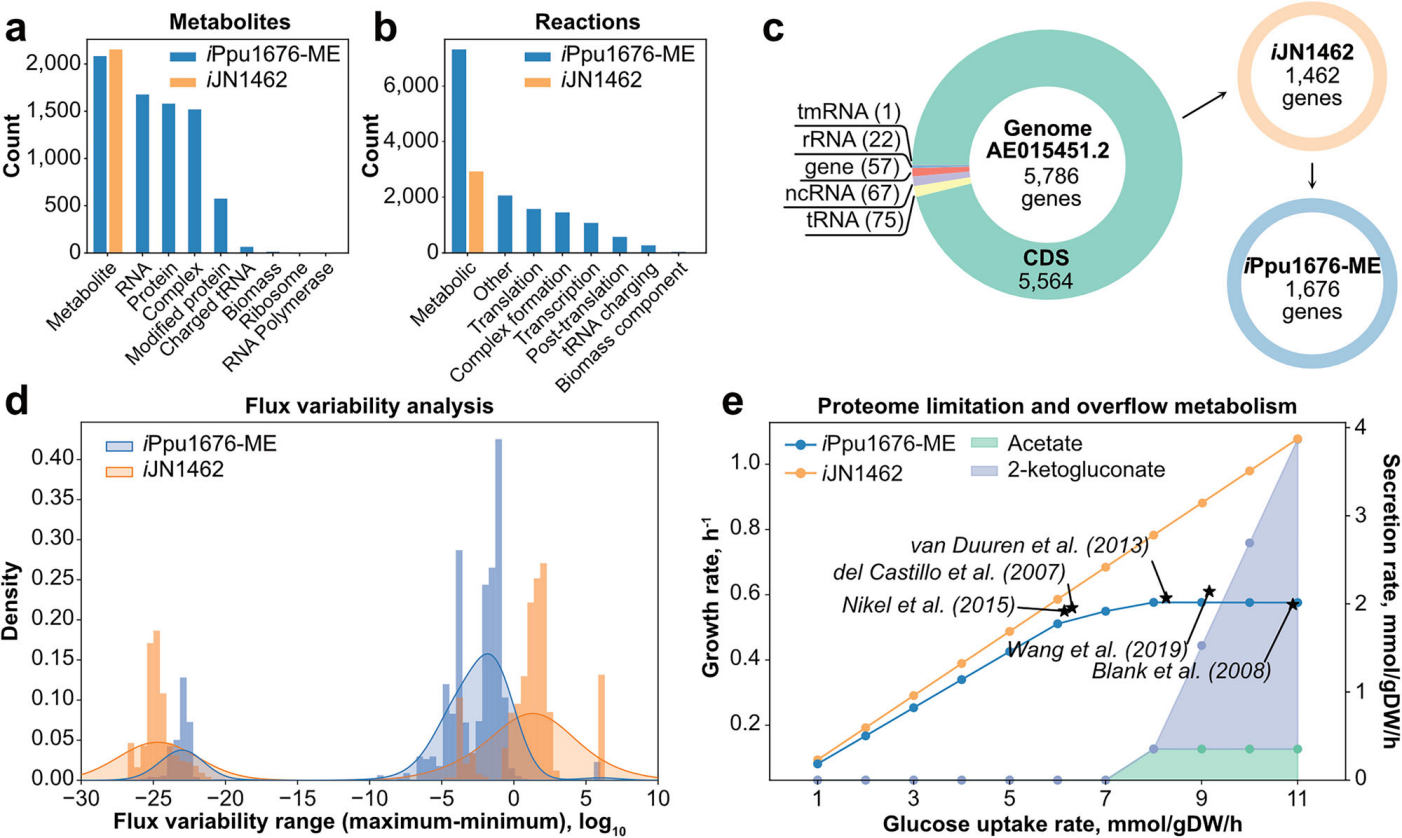
Properties and predictions of the ME-model of *P. putida* KT2440. **a** and **b** Breakdown of metabolites (**a**) and reactions (**b**) in *i*Ppu1676-ME as compared to the template M-model, *i*JN1462^1^. **c** Genome coverage of *i*Ppu1676-ME and *i*JN1462. **d** Comparison of flux variability analysis of the M- and ME-models of *P. putida* KT2440. The distribution of the flux ranges is bimodal due to the tolerance of the QuadMINOS solver of 10^−16^, below which fluxes can vary at a negligible level. **e** Prediction of the maximum growth rate of *P. putida* KT2440 by the ME-model as opposed to the overestimation in the M-model, compared to reported maximum growth rates^6,21–24^. Area plots show the predicted secretion rates of overflow metabolites acetate and 2-ketogluconate by the ME-model, which the M-model does not predict.

The ME-model of *Pseudomonas putida* KT2440, *i*Ppu1676-ME, was reconstructed based on a previous genome-scale metabolic model (M-model), *i*JN1462^1^. The gene expression machinery was integrated into the ME-model following available ME-model reconstruction protocols^12,18^. Protein complex stoichiometries, function, localization, translocation pathways, and transcriptional unit compositions were retrieved and mapped from the genome of *P. putida* KT2440 (AE015451.2^19^) as well as the strain-specific database in BioCyc^20^. *i*Ppu1676-ME consists of 7526 metabolites, 14,414 reactions, and 1676 genes. Compared to the original M-model template this represents an increase of 250% in metabolite, 392% in reaction, and 15% in gene coverage (Fig. 1a–c). The expression machinery (E-matrix) adds up to 5443 metabolites, including types of RNA (mRNA, tRNA, and rRNA), proteins with and without modifications, and complexes (Fig. 1a). In addition, the E-matrix contains 5040 reactions, including translation and modification, translocation, transcription, and tRNA charging (Fig. 1b). Finally, the added 214 genes in *i*Ppu1676-ME (Fig. 1c) correspond to mapped gene expression machinery including tRNA ligases, ribosomal proteins, RNA polymerase subunits, transcription factors, and protein modification machinery (including translocation machinery) (Supplementary Fig. 1).

As previously reported, the integration of the E-matrix provides the ME-model with a quantitative description of the biosynthetic cost of biochemical reactions^12^. Reaction fluxes are limited by the underlying gene expression cost, resulting in reduced flux variability^11^ and higher certainty in model predictions. The flux variability analysis of *i*Ppu1676-ME showed reduced flux ranges (Fig. 1e). The majority of the M-model ranges are above 10^0^, while ME-model ranges are mostly below it, increasing the certainty of predictions in the latter. Moreover, the ME-model readily recapitulates the maximum growth rate of *P. putida* KT2440 in a glucose-containing minimal medium (Fig. 1d), which has been reported at 0.58 (*σ* = 0.02) h^−1^ and at a glucose uptake rate of 8.15 (*σ* = 2.00) mmol/gDW/h from five different studies^6,21–24^. As opposed to the M-model, whose metabolic reactions are unconstrained by biosynthetic costs, the ME-model reaches proteome limitation when in nutrient excess and is capable of generating biologically relevant simulations without any additional constraints (Fig. 1d).

We further assessed the accuracy of intracellular flux predictions by contrasting them to previously published reports of metabolic flux analysis (MFA) in *P. putida* KT2440^25,26^. Notably, both M- (Supplementary File 1) and ME-models (Supplementary File 2) reproduce the overall activity of the core metabolism assessed in the experimental MFA studies. At a glucose uptake rate of 2.21 mmol/gDW/h^25^ (simulation constraints and flux distributions are provided in Supplementary Data 1), glucose is converted to 6-phospho-d-gluconate, which is then assimilated and conveyed to glycerol-3-phosphate, bypassing the upper half of glycolysis. Interestingly, there is minimal but nonzero flux through part of the pentose phosphate pathway predicted by both M- and ME-models and observed in MFA^26^. The second half of glycolysis is active and feeds into the tricarboxylic acid cycle, with all enzymatic steps being active^25,26^. However, only the M-model incorrectly predicted isocitrate lyase and malate synthase (the glyoxylate shunt) to be active, which were observed to be inactive in MFA^25,26^. Furthermore, we found that the ME-model correctly predicts the activity of pyruvate kinase^25,26^, which is inactive in the M-model simulations. In the latter, phosphoenolpyruvate is converted to pyruvate through dGTP:pyruvate 2-O-phosphotransferase.

Another improvement in ME-models is the prediction of proteome limitation, which leads to the mechanistic prediction of overflow metabolites in ME-models^15^. In *i*Ppu1676-ME, this leads to predicting 2-ketogluconate and acetate secretion in excess of glucose (Fig. 1e). While 2-ketogluconate is a known secreted metabolite by *P. putida*, acetate was predicted as a minor by-product, which has been shown in oxygen-limited conditions^22,27^. Notably, *i*Ppu1676-ME maintains the same 85% accuracy in gene essentiality prediction^1^ of 54 metabolic gene knockout strains in M9 minimal medium^28^.

### Multi-omic data reveal translational prioritization in *P. putida* KT2440

Translational efficiency (TE) refers to the rate of protein synthesis per unit of mRNA transcript^29^. The translation of a group of genes is said to be prioritized if their TE (see the “Methods” section) is high relative to other genes^29^. Translational prioritization readily informs the resource allocation strategies of an organism^30–32^. It can be used to infer specific objectives^30,33^, such as maximizing growth or uptaking a substrate. Thus, we aimed to interrogate the proteome allocation of *P. putida* and its translational prioritization using RNA-Seq and Ribo-Seq. Then, we contrasted it with the predictions by *i*Ppu1676-ME.

**Fig. 2:**
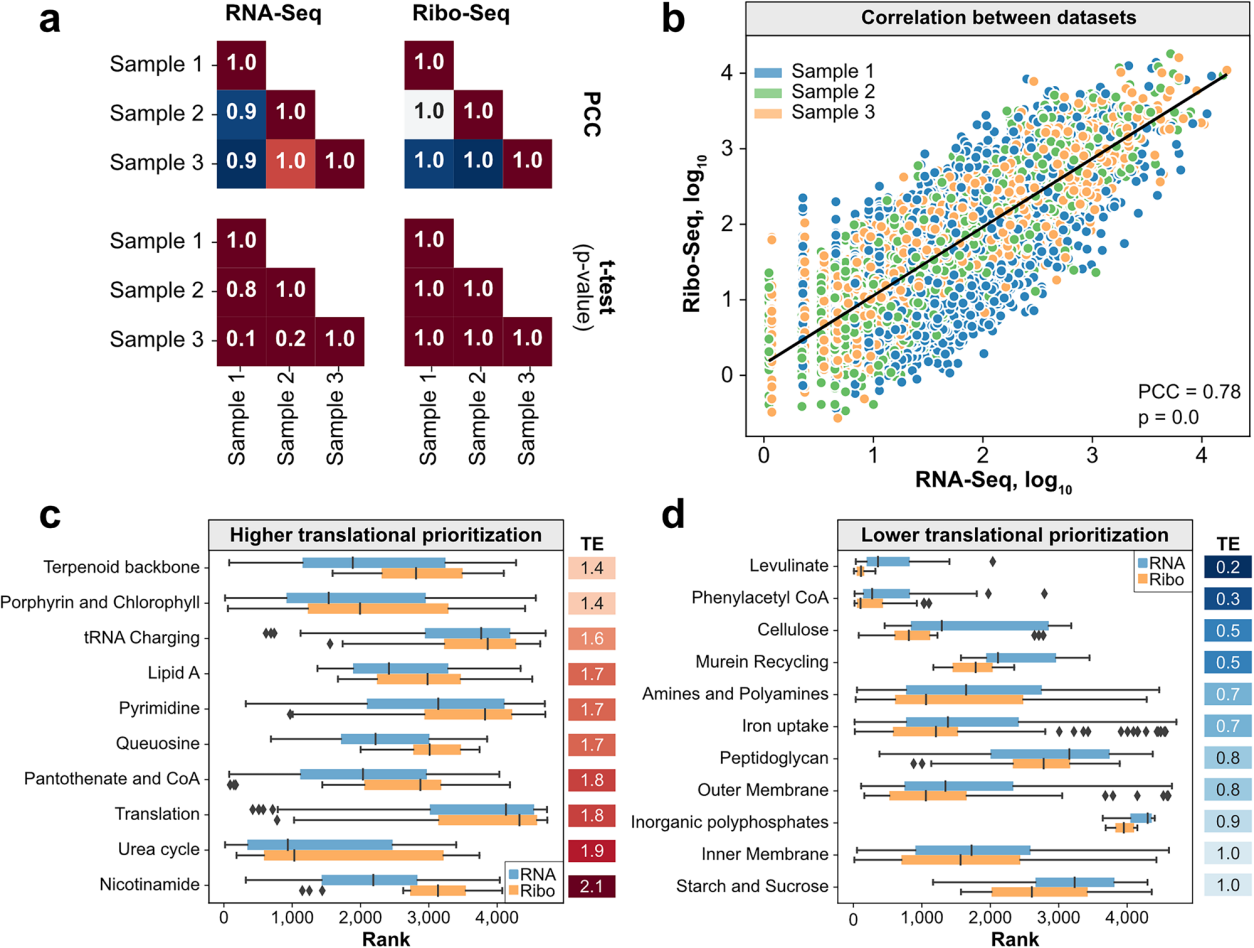
Multi-omic data of *P. putida* KT2440 growth in glucose. **a** Pearson correlation coefficient (PCC) of and two-tailed *t*-test (*p*-values) for the difference in mean of the CPM distributions in the RNA-Seq and Ribo-Seq replicates. Strong correlation and no significant mean difference across replicates indicate there are no replicate outliers. **b** Correlation of RNA-Seq and Ribo-Seq CPM in the three samples. **c** Metabolic pathways with higher rank in the Ribo-Seq dataset against the RNA-Seq dataset (*p* < 0.05 as calculated by the right-tailed Mann–Whitney *U*-test). Translational efficiencies (TEs) are shown for reference. **d** Metabolic pathways with lower rank in the Ribo-Seq dataset against the RNA-Seq dataset (*p* < 0.05 as calculated by the left-tailed Mann–Whitney *U*-test).

*P. putida* was grown in glucose-containing M9 minimal medium in three biological replicates, and paired RNA-Seq and Ribo-Seq were performed. The three samples yielded a wide range of gene activity in both datasets with four and six orders of magnitude differences, as observed in the raw read counts from RNA-Seq (Supplementary Fig. 2a) and Ribo-Seq (Supplementary Fig. 2b), respectively, reinforcing the disparity in resource allocation throughout the genome. In order to discard technical artifacts^34^ as a confounding factor, we performed two-tailed *t*-tests (*p* < 0.05) and calculated Pearson correlation coefficients of the replicates. No significance in the means of the read count distributions was observed, and all replicates were very strongly correlated (PCC > 0.9) in both RNA-Seq and Ribo-Seq (Fig. 2a).

When contrasting across datasets, RNA-Seq and Ribo-Seq counts-per-million (CPM) show significant correlations, with a PCC of 0.78 (*p* = 0.0) in the three samples (Fig. 2b). Despite the significant correlation at the whole-dataset level, there are variations in the resource allocation at the pathway level in the transcriptome and the translatome, which shows differential translational prioritization across the genome of *P. putida*. Thus, we assessed whether metabolic pathways were observed with high or low translational prioritization. We measured the translational prioritization of a pathway using the average TE of its associated genes (see the “Methods” section) and calculated the significance of the prioritization through a one-tailed Mann–Whitney *U* (MW*U*) test. As part of the MW*U* test, we sorted and ranked the genes in the RNA-Seq and Ribo-Seq datasets and contrasted if their ranks differed significantly in either dataset. A high TE and a low right-tailed MW*U* test *p*-value for a significant increase in rank in the Ribo-Seq dataset (*p* < 0.05) support high translational prioritization.

A right-tailed Mann–Whitney *U* test revealed that there were ten metabolic pathways with significantly higher ranks in the Ribo-Seq dataset (*p* < 0.05), which can be evidenced by the calculated translational efficiencies (TE)^30,35,36^ between 1.4 and 2.1 (Fig. 2c). Notably, the highest prioritization (highest TE) was calculated for nicotinamide biosynthesis (TE = 2.1, *p* = 5.38e−4), which produces the essential cofactor NAD^+^. Other core metabolic pathways, such as urea cycle (TE = 1.9, *p* = 0.02), pyrimidine biosynthesis (TE = 1.7, *p* = 5.04e−3), lipid A biosynthesis (TE = 1.7, *p* = 0.02), and queuosine biosynthesis (TE = 1.7, *p* = 0.006), which are directly associated with cell proliferation^37^, showed significant prioritization. As expected, there was significant prioritization of gene expression, including translation (TE = 1.8, *p* = 0.03) and tRNA charging (TE = 1.6, *p* = 0.04).

On the other hand, 11 metabolic pathways had significantly lower ranks as calculated by a left-tailed Mann–Whitney *U* test (*p* < 0.05). The lowest prioritization was shown for pathways less necessary for growth in glucose, namely levulinate metabolism (TE = 0.2, *p* = 7.02e−4), phenylacetyl-CoA catabolon (TE = 0.3, *p* = 0.03), cellulose metabolism (TE = 0.5, *p* = 0.02), and starch and sucrose metabolism (TE = 1.0, *p* = 0.01). Membrane-associated pathways, such as inner membrane transport (TE = 1.0, *p* = 0.02), outer membrane transport (TE = 0.8, *p* = 8.5e−3), iron uptake (TE = 0.7, *p* = 0.03), peptidoglycan biosynthesis (TE = 0.8, *p* = 0.04), and murein recycling (TE = 0.5, *p* = 0.01), showed low translational prioritization. It is worth noting that a TE equal to 1.0 can still mean an overall lower prioritization due to the variation in ranks in both datasets, such is the case for starch and sucrose metabolism and inner membrane transport (Fig. 2d).

### The ME-model recapitulates optimal proteome allocation

**Fig. 3:**
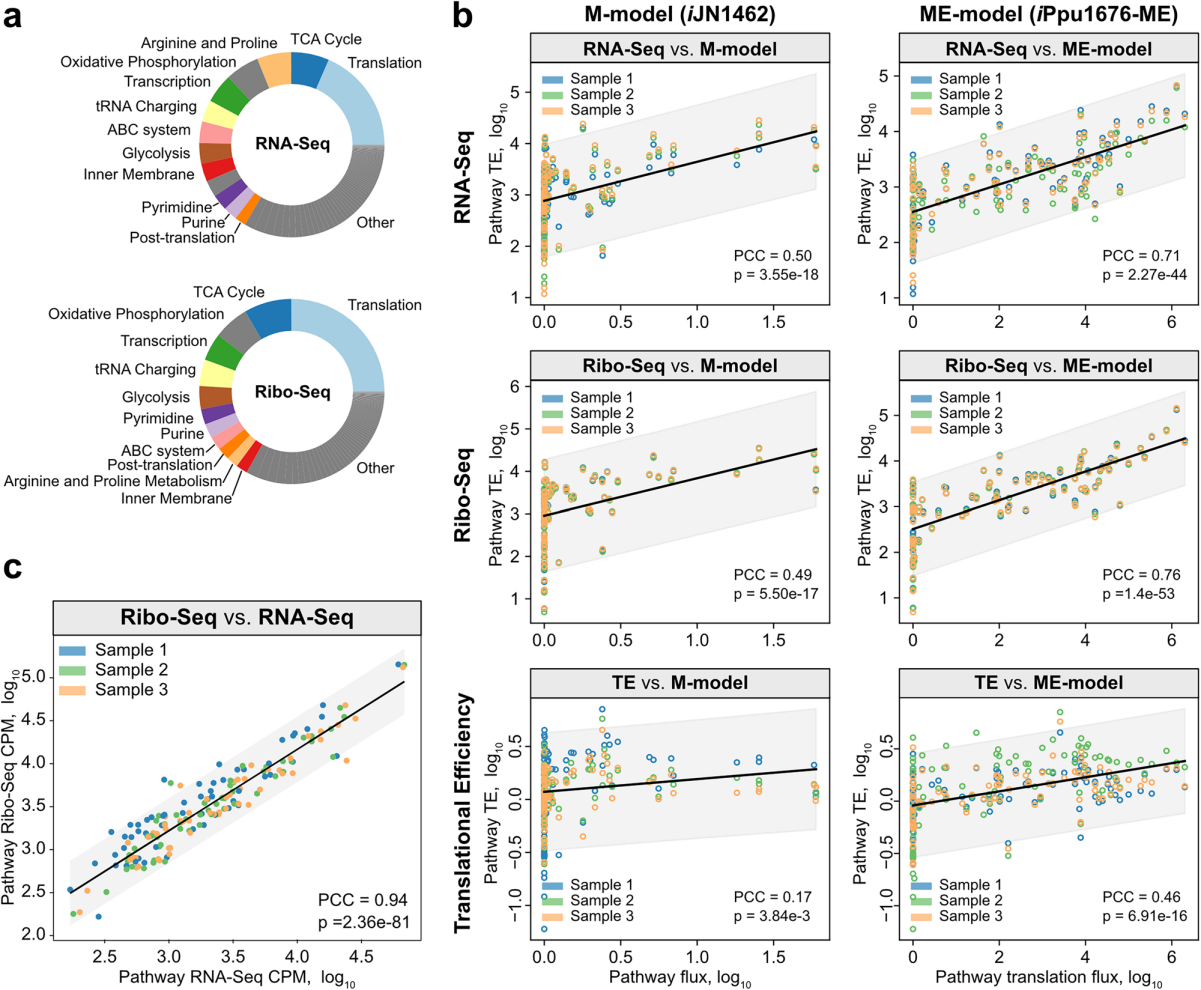
Correlation between model predictions and multi-omics at the pathway level. **a** and **b** Accumulated counts per million (CPM) for each pathway in the RNA-Seq (**a**) and the Ribo-Seq (**b**) datasets. Only the top 10 largest contributing pathways in either dataset are highlighted. **b** Correlation between Ribo-Seq and RNA-Seq CPM at the pathway level. **c** Correlation between multi-omics and M- (*i*JN1462) or ME-model (*i*Ppu1676-ME) predictions at the pathway level.

Our translational prioritization analysis highlighted significantly higher TEs for growth-required pathways, both metabolic and gene expression-related (Fig. 2c, d). Most of the transcriptome and translatome are allocated for these pathways (Fig. 3a), led by translation and followed by energy and biomass production. However, multi-omic data does not provide a quantitative understanding of the metabolic and gene expression rates. Predictive metabolic models are used to attain this understanding. Thus, here, we assessed the improved performance of the ME-model of *P. putida* KT2440 over the M-model when contrasting against the expression levels inferred from multi-omics (simulation constraints and flux distributions are provided in Supplementary Data 2).

We compared M- and ME-model predictions of cumulative pathway-level fluxes of *P. putida* KT2440 against pathway-level expression from RNA-Seq, Ribo-Seq, and the calculated TE from them. The ME-model significantly outperformed the M-model in all cases (Fig. 3b). As a reference, pathway-level RNA-Seq and Ribo-Seq are correlated with a PCC of 0.94 (Fig. 3c). In the M-model, several subsystems were predicted with low flux, causing relevant data points to be more sparse. The PCCs were 0.50 for RNA-Seq (*p* = 3.55e−18) and 0.49 for Ribo-Seq (*p* = 5.50e−17), which signifies an existent but weak correlation between both datasets. On the other hand, the ME-model showed stronger positive correlations with PCCs of 0.71 (*p* = 2.27e−44) and 0.76 (*p* = 1.40e−53) for RNA-Seq and Ribo-Seq, respectively. Notably, despite the stronger correlation between the ME-model predictions and RNA-Seq and Ribo-Seq, there was only a weak positive correlation for TE (PCC = 0.46). Therefore, the predicted expression fluxes by the ME-model are predictive of the observed transcriptome and translatome but not of the TE. Thus, the predictive capability of *i*Ppu1676-ME to recapitulate proteome allocation of *P. putida* KT2440 showcases its potential to be further used to interrogate and optimize the resource allocation in this organism.

## Discussion

*Pseudomonas putida* KT2440 has broad potential for biotechnological applications^1,2^, and several studies have been focusing on optimizing culture conditions, growth, and metabolic capabilities for this bacterium. While bioinformatics tools and databases have informed potential pathways in *P. putida*^38,39^, only predictive metabolic models can quantitatively estimate improvements in metabolism, growth, product rates, and yields^40–42^. Similar to previous studies using ME-models, we here showed the prediction of by-product secretion^11^, overflow metabolism^15^, and prediction of proteome allocation^16^. While previous ME-model reconstructions have widely proven prediction improvements, multi-omics datasets (RNA-Seq and Ribo-Seq) have not been integrated or contrasted with these simulations. Our translational prioritization analysis showed significantly high TE for queuosine biosynthesis alongside core biomass precursor biosynthetic pathways. Interestingly, this pathway has been reported as a cell division regulator in other bacteria^37^. On the other hand, transport and other membrane-associated functions were found to have low translational prioritization.

The ME-model for *P. putida* KT2440, *i*Ppu1676-ME, showed significant improvements in the predictive capabilities of transcriptome and translatome over the template M-model, *i*JN1462^1^. *i*Ppu1676-ME achieved an outstanding PCC of 0.71 against RNA-Seq and 0.76 against Ribo-Seq. As a reference, the multi-omics model and analytics (MOMA), a semi-supervised machine learning pipeline, achieved PCCs between 0.58 and 0.85 with RNA-Seq in *E. coli* across 16 strains^43^. It is worth noting that the higher agreement of Ribo-Seq and the predicted proteome allocation by the ME-model underscores the precision of this sequencing technology in identifying the metabolic goal of an organism^30^. In addition, it highlights the ability of a ME-model to predict optimal proteome allocation in a metabolically diverse organism such as *P. putida* KT2440^1,2^.

Some limitations affect our analysis. Determination of the active proteome is challenging through sequencing technologies due to various technical limitations. For example, RNA degradation affects measurements by RNA-Seq^44^, and RNA transcription trends do not always carry over to translation due to translational prioritization effects^29,30^. On the other hand, direct measurement of the proteome through proteomics is hindered by the difficulty of whole-proteome determinations and the inherent noise in mass spectrometry data^45^. On the other hand, Ribo-Seq provides an accurate representation of translation in vivo^29,30^. While it cannot detect protein stability, modification, and folding^46^, it has been shown to provide a genome-scale understanding of translational prioritization^29,30^. Furthermore, we noticed variation between the replicates, which can be due to the inherent flexibility of the metabolism of *P. putida*^1,2^. However, the correlation was strong enough (PCC > 0.9) to ensure there were no significant outliers in the replicates. Another limitation of this study is that metabolic models, and thus ME-models, are limited to modeling enzymes with either a metabolic or a gene expression function^12^. However, there is a fraction of the proteome that can have an alternative function, which can be structural or still unknown. For example, this fraction has been estimated to be approximately 36% of the proteome in *E. coli*^12^, and it might be the cause of some of the unexplained variances in our comparison between model predictions and Ribo-Seq.

Here, we provide a modeling framework alongside multi-omic datasets that were not available to date, yielding an important resource for further understanding the translational resource allocation in this industrially relevant microorganism. Overall, we envision that the ME-model and multi-omic resources brought forward in this work will serve as powerful tools for the metabolic engineering and optimization of *P. putida* KT2440.

## Methods

### Reconstruction and simulation of the ME-model

The ME-model of *P. putida* KT2440, *i*Ppu1676-ME, was reconstructed using coralME^18^, with the available genome AE015451.2^19^, M-model (*i*JN1462^1^), and BioCyc^20^-derived annotation files as inputs. Input manual curation files of *i*Ppu1676-ME are explained in Table 1. The code and scripts in this work were developed and run in Python 3.10, COBRApy^47^ version 0.26.3, and coralME version 1.1.5. Simulations were performed using the Quad MINOS software courtesy of Prof. Michael A. Saunders at Stanford University^48^. The Quad MINOS solver was compiled under Ubuntu 22.04 with gfortran version 5 and Python 3.10 (pip 22.3.1, wheel 0.38.4, numpy 1.21.6, scipy 1.7.3, cython 0.29.32, and cpython 0.0.6). Flux distributions were calculated with a binary search algorithm that looks for the maximum possible growth rate that is feasible.

**Table 1:** Input and manual curation files and their description

Name	Description	Type
genome.gb	Genome file of *P. putida* KT2440 (AE015451.2^19^). Obtained from NCBI.	Input file
m_model.json	M-model file of *i*JN1462. Obtained from publication^1^.	Input file
RNAs.txt	All RNAs, types (mRNA, tRNA, rRNA, etc.), and their products. Obtained from BioCyc^20^.	Input file
TUs.txt	Transcriptional units, their locations, and compositions. Obtained from BioCyc^20^.	Input file
genes.txt	All genes, types (CDS, RNA, etc.), and their products. Obtained from BioCyc^20^.	Input file
proteins.txt	All proteins and their annotation. Obtained from BioCyc^20^.	Input file
sequences.fasta	All genes and their nucleotide sequences. Obtained from BioCyc^20^.	Input file
me_metabolites.txt	Conversion of non-metabolic metabolites in the M-model into their respective representations in the ME-model. For example, “ACP_c” in the ME-model becomes G1G01-2027-MONOMER_mod_pan4p(1).	Manual curation file
subreaction_matrix.txt	Modifications and additions to ME-model subreactions. Most of these are ultimately incorporated in complex formation or tRNA charging reactions. For example, “mod_acetyl_c” re-defines the stoichiometry for the acetylation modification of proteins.	Manual curation file
reaction_corrections.txt	Modifies reactions in the M-model based on manual inputs in reaction_corrections.txt.	Manual curation file
metabolite_corrections.txt	Modifies metabolites in the M-model before ME-model building using manual inputs in metabolite_corrections.txt.	Manual curation file
peptide_compartment_and_pathways.txt	Adds protein locations and translocation pathways to an Organism instance from peptide_compartment_and_pathways.txt.	Manual curation file
translocation_multipliers.txt	Defines the number of pores required for protein translocation using data from translocation_multipliers.txt.	Manual curation file
lipoprotein_precursors.txt	Adds lipoprotein precursors to an Organism instance using manual inputs from lipoprotein_precursors.txt.	Manual curation file
cleaved_methionine.txt	Marks proteins for N-terminal methionine cleavage in the ME-model using cleaved_methionine.txt.	Manual curation file
protein_corrections.txt	Modifies or adds protein complexes in the ME-model using inputs from protein_corrections.txt.	Manual curation file
sigma_factors.txt	Adds sigma factors for transcription regulation in an Organism instance using sigma_factors.txt.	Manual curation file
rho_independent.txt	Marks genes with rho-independent transcription termination using rho_independent.txt.	Manual curation file
rna_degradosome.txt	Defines the composition of the RNA degradosome using rna_degradosome.txt.	Manual curation file
rna_modification.txt	Adds RNA modification enzymes for rRNA or tRNA modifications in the ME-model using rna_modification.txt.	Manual curation file
post_transcriptional_modification_of_RNA.txt	Defines RNA genes undergoing modifications using post_transcriptional_modification_of_RNA.txt.	Manual curation file
enzyme_reaction_association.txt	Associates enzymes with reactions in the ME-model using enzyme_reaction_association.txt.	Manual curation file
reaction_matrix.txt	Defines reactions to be added to the ME-model using reaction_matrix.txt.	Manual curation file
orphan_and_spont_reactions.txt	Marks reactions as orphan or spontaneous in the ME-model using orphan_and_spont_reactions.txt.	Manual curation file
subsystem_classification.txt	Classifies subsystems for Keff estimation in the ME-model using subsystem_classification.txt.	Manual curation file
translocation_pathways.txt	Defines translocation pathways and their machinery using translocation_pathways.txt.	Manual curation file
lipid_modifications.txt	Defines lipid modification enzymes using lipid_modifications.txt.	Manual curation file
ribosomal_proteins.txt	Defines ribosome composition using ribosomal_proteins.txt.	Manual curation file
ribosome_subreactions.txt	Defines ribosome subreactions using ribosome_subreactions.txt.	Manual curation file
generic_dict.txt	Defines generic enzyme components using generic_dict.txt.	Manual curation file
amino_acid_trna_synthetase.txt	Associates amino acids with tRNA synthetases using amino_acid_trna_synthetase.txt.	Manual curation file
peptide_release_factors.txt	Defines peptide release factors using peptide_release_factors.txt.	Manual curation file
initiation_subreactions.txt	Defines translation initiation subreactions using initiation_subreactions.txt.	Manual curation file
elongation_subreactions.txt	Defines translation elongation subreactions using elongation_subreactions.txt.	Manual curation file
termination_subreactions.txt	Defines translation termination subreactions using termination_subreactions.txt.	Manual curation file
transcription_subreactions.txt	Defines transcription subreactions and their associated enzymes using transcription_subreactions.txt.	Manual curation file
special_trna_subreactions.txt	Defines special tRNA subreactions such as tRNA-Sec synthesis using special_trna_subreactions.txt.	Manual curation file
special_modifications.txt	Defines special protein modifications in the ME-model using special_modifications.txt.	Manual curation file
excision_machinery.txt	Defines excision machinery using excision_machinery.txt.	Manual curation file
folding_dict.txt	Defines protein folding pathways using folding_dict.txt.	Manual curation file

Computations were performed on a 64-bit Ubuntu 22.04.3 LTS (Jammy Jellyfish); AMD Ryzen 9 7900X@4.70 GHz (12 cores, 24 threads); 4 × 32 GB 6000 MHz DDR5 RAM. It is worth noting that *i*Ppu1676-ME is one of the largest ME-models available. As a reference, the *E. coli* ME-model contains 1678 genes^12^. As such, optimizing *i*Ppu1676-ME typically takes five minutes in the computer described here. This is several orders of magnitude longer than the associated M-model, *i*JN1462^1^ (~72 ms). While this computation time still allows for most analyses performed in metabolic modeling, it can hinder its application in large-scale sampling of conditions, e.g., simulating a bioreactor.

### Gene essentiality predictions

Gene essentiality was predicted in *i*Ppu1676-ME by closing (setting upper and lower bounds to zero) the translation reaction of a gene and testing for ME-model feasibility. Feasibility is tested at a growth rate of 10^−3^ with the QuadMINOS solver in quad-precision and a tolerance of 10^−16^.

### Flux variability analysis

Flux variability analysis (FVA) was performed using the built-in method *model.fva()* in coral ME. In order to generate comparable datasets between the M- and the ME-models, we cast the M-model into an ME-model instance (function *from_cobra()*). The FVA in both models was performed using the QuadMINOS solver in quad-precision and a tolerance of 10^−16^.

### Culture and sequencing of *P. putida* KT2440

A preculture of *Pseudomonas putida* KT2440 was grown in 5 mL of liquid 1× M9 medium (Sigma Aldrich, M6030) with 30 mM glucose under oxic conditions at 37 °C for 2 days. The optical density at 600 nm (OD_600_) was measured to assess carrying capacity using the Molecular Devices SpectraMax M3 Multi-Mode Microplate Reader (VWR, cat # 89429-536). The preculture was diluted in triplicate to an OD_600_ of 0.1 in 25 mL of 1x M9 medium and incubated under oxic conditions at 37 °C in a shaking incubator, with optical density measured hourly. Samples were harvested by centrifugation and pellets were saved for multi-omics analysis as detailed below (RNA-, and Ribo-Seq).

### Transcriptomic (RNA-Seq) sample preparation

RNA was extracted from *P. putida* replicates using the RNeasy mini kit (Qiagen), with rRNA removal performed using the QIAseq FastSelect-5S/16S/23S kit (Qiagen). RNA-Seq libraries were constructed using the KAPA RNA HyperPrep kit (Roche) and barcoded with TruSeq indexes (Illumina). Amplification was monitored in real-time using SYBR-Green and halted upon reaching the amplification plateau.

### Translatomic (Ribo-Seq) sample preparation

The preparation of Ribo-Seq samples utilized a protocol adapted from previously described methods designed for axenic bacterial cultures^33,49^. Briefly, the bacterial cultures were treated with chloramphenicol and pelleted. At this point, we modified this protocol to address the chloramphenicol resistance of *Pseudomonas putida* KT2440 by resuspending the pellet in RNAlater and flash-freezing in liquid nitrogen. Samples were thawed, pelleted, and RNAlater removed, before proceeding to mechanical bacterial lysis. Bacterial lysis was performed in the presence of a lysis solution containing additional chloramphenicol and Guanosine-5′-[(β,γ)-imido] triphosphate (GMPPNP) to inhibit protein elongation. Lysates were treated with MNase and DNase to digest nucleic acids that were not protected by ribosomes. Monosomes were recovered using RNeasy mini spin size-exclusion columns (Qiagen) and RNA Clean & Concentrator-5 kit (Zymo). rRNA was removed with the QIAseq FastSelect-5S/16S/23S kit (Qiagen), and MetaRibo-Seq libraries were constructed using the NEBNext Small RNA Library Prep set for Illumina. Amplification was followed in real-time with SYBR-Green and stopped upon plateau plateau, and PCR products were purified with the Select-a-size DNA Clear & Concentrator kit (Zymo).

### Sequencing

Library quantity and average size were assessed with the 4200 TapeStation System (Agilent). Library concentrations were quantified using the Qubit dsDNA HS Assay kit and QuBit 2.0 Fluorometer (Invitrogen). Sequencing was performed by UCSD IGM on the Illumina NovaSeq S4, PE100 platform with a minimum sequencing depth of 50 million reads for transcriptomic samples and 100 million reads for translatomic samples.

### Sequence alignment and post-processing of RNA-Seq and Ribo-Seq

Paired-end read sequencing files from RNA-Seq and Ribo-Seq (FASTQ format) were processed using Python 3.7. Reads were trimmed using trim_galore version 0.6.10 (Cutadapt version 2.6)^50^. Reads were aligned to the genome of *P. putida* KT2440 using bowtie2 version 2.2.5^51^. For Ribo-Seq, single-end read sequence alignment was performed since read length is short enough that both directions are redundant. For RNA-Seq, reads aligning to ribosomal RNA were discarded. Raw read counts were estimated using Woltka version 0.1.5^52^, with the built-in function “classify”. Finally, cross-sample analyses in this work were performed using the counts per million (CPM) normalization of read counts, as shown in Eq. (1).

1 $$CPM=\frac{Raw\,read\,count}{\sum Raw\,read\,counts\,in\,sample}\,\ast {10}^{6}$$

### Statistical analysis of translational prioritization from multi-omics

Translational prioritization was inferred from calculating translational efficiency (TE) (Eq. (2))^30,35,36^, and significance was calculated using the Mann–Whitney *U* test. Higher translational prioritization was determined if TE ≥ 1.0 and *p* < 0.05 in a right-tailed Mann–Whitney *U* test, while lower was determined if TE ≤ 1.0 and *p* < 0.05 in a left-tailed Mann–Whitney *U* test. The test was performed using scipy.stats.mannwhitneyu package of scipy version 1.11.1^53^.

2 $$TE=\frac{Ribo-Seq\,CPM}{RNA-Seq\,CPM}$$

### Benchmarking analysis of model predictions with multi-omics

Benchmarking of the models (M- and ME-models) predictions was performed with a correlative analysis between the pathway activity inferred from RNA-Seq and Ribo-Seq and the predicted pathway fluxes in the M-model (*i*JN1462^1^) and the ME-model (*i*Ppu1676-ME). For RNA-Seq and Ribo-Seq, gene-level CPM data were grouped and summed by annotated subsystem in the ME-model. For M-model predictions, the reaction fluxes were grouped and summed by annotated subsystem in the M-model. For ME-model predictions, protein translation fluxes were grouped and summed by annotated subsystem in the M-model. We then log-transformed the fluxes as log_10_(*f* + 1), where *f* is the combined flux of a subsystem. The data in all three samples was used in the regression to maintain maximal statistical power. Then, linear regression was performed with the statsmodels version 0.14.1, which returned the regressed linear model and 95% confidence interval. The Pearson correlation coefficient (PCC) and significance (*p*-value) were calculated using scipy.stats.pearsonr of scipy version 1.11.1.

## Supplementary information

Supplementary information

Supplementary File 1

Supplementary File 2

Supplementary Data 1

Supplementary Data 2

## Data Availability

Data is provided within the manuscript and in supplementary information. Additionally, all data in this work, except for the raw sequencing files, are available in the GitHub repository at https://github.com/jdtibochab/pputidame. The code and data have been deposited in Zenodo (10.5281/zenodo.14984894). Raw RNA-Seq and Ribo-Seq files have been deposited in the Sequence Read Archive (SRA) under BioProject PRJNA1238387.

## Code availability

All code used in this work is available in the GitHub repository at https://github.com/jdtibochab/pputidame. The code and data have been deposited in Zenodo (10.5281/zenodo.14984894).
