# Supplementary material for: Model of metabolism and gene expression predicts proteome allocation in Pseudomonas putida
Source: NPJ Syst Biol Appl. 2025 May 24;11:55. doi: 10.1038/s41540-025-00521-1 (PMC12103522; doi:10.1038/s41540-025-00521-1)
Supplement: Supplementary file 1 — Supplementary information [file 41540_2025_521_MOESM1_ESM.pdf]

Supplementary Information for

Model of metabolism and gene expression predicts proteome allocation in *Pseudomonas putida*

Juan D. Tibocha-Bonilla *et al.*

*Corresponding author. Email: (Karsten Zengler) kzengler@ucsd.edu

This PDF file includes:

Supplementary Figures 1 to 2

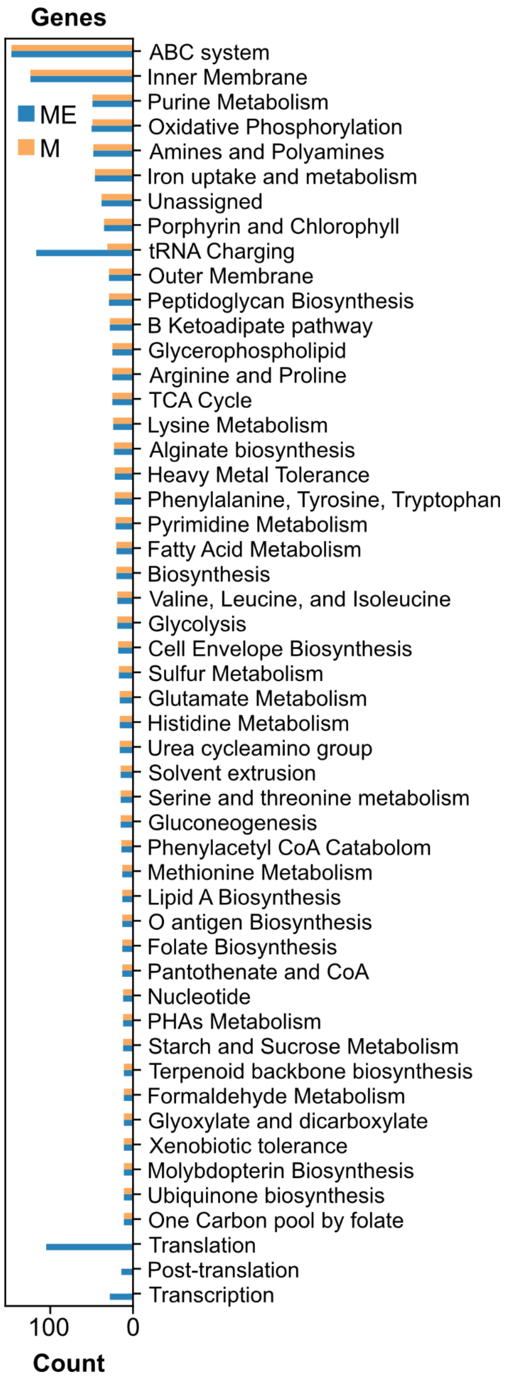

Supplementary Figure 1. Breakdown of genes in their respective annotated pathways in *i*Ppu1676-ME and the template M-model, *i*JN1462.

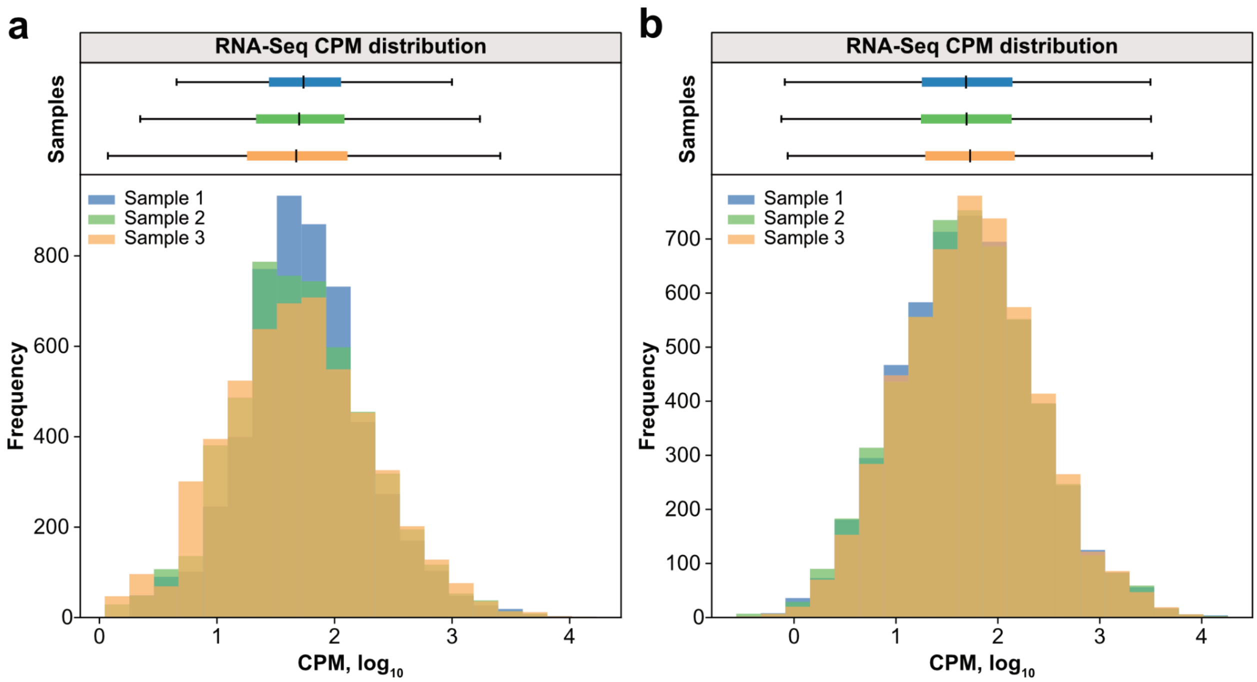

Supplementary Figure 2. Multi-omic data (CPM) of *P. putida* KT2440 growth in glucose. a, Distribution of RNA-Seq raw read counts in the three biological replicate samples. **b,** Distribution of Ribo-Seq raw read counts in the three samples.
